# Sublethal effects of salinity and temperature on non-native blue catfish: Implications for establishment in Atlantic slope drainages

**DOI:** 10.1371/journal.pone.0244392

**Published:** 2020-12-29

**Authors:** Vaskar Nepal, Mary C. Fabrizio

**Affiliations:** Virginia Institute of Marine Science, William & Mary, Gloucester Point, Virginia, United States of America; Uppsala Universitet, SWEDEN

## Abstract

The distribution and further range expansion of non-native blue catfish *Ictalurus furcatus* in coastal waters throughout the United States Atlantic slope depend, in part, on the salinity tolerance of the fish. However, temperature-mediated sublethal effects of increased salinities on blue catfish biology are not yet known. We assessed the effects of salinity and temperature on growth, body condition, body composition and food consumption of juvenile blue catfish in a controlled laboratory experiment. Temperature and salinity had an interactive effect on blue catfish biology, although most fish survived 112 days in salinities up to 10 psu. At salinities ≤7 psu, mean growth rate, body condition and consumption rates were higher at 22°C than at 12°C. Mean consumption rates declined significantly with increasing salinities, yet, salinities ≤7 psu were conducive to rapid growth and high body condition, with highest growth and body condition at 4 psu. Fish at 10 psu exhibited low consumption rates, slow growth, low body condition and lower proportions of lipids. Habitats with hyperosmotic salinities (>9 psu) likely will not support the full lifecycle of blue catfish, but the fish may use salinities up to 10 psu for foraging, dispersal and even growth. Many oligohaline and mesohaline habitats in U.S. Atlantic slope drainages may thus be vulnerable to establishment of invasive blue catfish, particularly given the increasing temperatures as a result of climate warming.

## Introduction

Biological invasions can cause major conservation, economic and human health issues in recipient ecosystems [1]. A classic example is Nile perch *Lates niloticus*, which contributed to the extinction of over 200 species of endemic cichlid fishes from Lake Victoria, after it was introduced into the lake to create a novel fishery [2]. Unsurprisingly, prevention of such catastrophic impacts due to invasive species is a priority for governments throughout the world, prompting policies to prevent the introduction of non-native species, to manage existing invasive species, and to minimize overall negative impacts of invasive species. An invasive species of increasing concern in Atlantic slope rivers of the United States is the blue catfish *Ictalurus furcatus* [3]. This freshwater fish, native to large Midwestern rivers, was introduced in tidal freshwater portions of the James, York and Rappahannock rivers in the Chesapeake Bay region during the 1970s and 1980s to create a recreational fishery [4]. Since then, the fish has expanded in range both within the tidal rivers where they were introduced and into most other tidal rivers throughout the Chesapeake Bay [4, 5]. Similar introductions have resulted in the establishment of non-native blue catfish populations in many tidal rivers along the Atlantic coast between Georgia and Delaware and in the Gulf of Mexico drainage in Florida [6]. In some of these systems, blue catfish densities are high, and this species may be numerically dominant in the catch of fisheries-independent surveys [7]. In addition, the generalist, opportunistic feeding behavior of blue catfish is likely impacting native species negatively via competition and predation [4, 8]. As such, resource managers in the Chesapeake Bay region are interested in managing blue catfish populations to limit further range expansion of the species and to decrease its negative impacts on native ecosystems [3].

The potential distribution of a species is determined by the species’ physiological constraints, which define its fundamental niche [9]. For blue catfish in the coastal rivers of the eastern U.S., salinity tolerance may limit its range expansion. Most freshwater fishes are unable to penetrate oligohaline (0–5 psu [practical salinity units] salinity) and mesohaline (5–18 psu salinity) environments in estuaries due to low physiological tolerance to elevated salinities or to biotic interactions such as competition with or predation from marine species [10]. In particular, hyperosmotic salinities (i.e., >9 psu) are expected to be uninhabitable by freshwater fishes due to the inability of fish to rearrange their osmoregulatory processes. Yet, blue catfish maintain native populations in oligohaline and mesohaline regions of coastal rivers in the southern United States, with fish captured most frequently at salinities <3.7 psu, but also at salinities up to 11.4 psu [11]. The species occurs in Atlantic slope rivers, where is it considered non-native; here, blue catfish are captured at salinities as high as 21.8 psu [12]. The ability to establish populations in high salinity environments could potentially increase the overall population size, connectivity and ultimately, the negative impacts of this species on estuarine organisms. Other non-native freshwater species such as pike *Esox lucius* and rainbow trout *Oncorhynchus mykiss* use brackish waters for reproduction and foraging, as migration corridors to new habitats or to avoid stressful abiotic conditions [13, 14]. The ability of blue catfish to establish populations in high salinity habitats is a concern among resource managers.

Juvenile blue catfish have a relatively high tolerance to acute, short-term increases in salinity, potentially allowing this invasive fish to exploit mesohaline environments for dispersal and range expansion throughout the Chesapeake Bay and into the Delaware Bay watershed [5]. The long-term effects of increased salinity on blue catfish biology, however, are not clear. Abass et al. [15] reported maximum survival and growth of hatchery-spawned larval blue catfish at sodium chloride concentrations of 3 ppt (parts per thousand), but 100% mortalities at salinities ≥6 ppt. These results, though useful as a general indication of the salinity tolerance of blue catfish, may not be readily applicable to wild fish [16]. Accurate projections of estuarine habitat use by blue catfish, therefore, require knowledge of sublethal impacts of salinity conditions on vital rates of fish.

Effects of salinity on physiological processes and vital rates of a fish depend on water temperature [17]. This is particularly important for fishes in temperate estuaries such as Chesapeake Bay where salinity and temperature vary annually and seasonally. Optimal habitats for a fish are, therefore, likely to change seasonally and annually in such environments. The quality of specific salinity and temperature conditions to a fish can be inferred by studying growth rates, body condition and energy reserves at those conditions: in suboptimal environments, fishes grow slowly and have low body condition and energy reserves, which together signify poor health [18]. Controlled experiments assessing these traits at various biologically relevant salinity and temperature conditions can inform predictions about the general health, well-being and invasion potential of blue catfish in Chesapeake Bay and other non-native estuarine habitats. Inferences could also be drawn regarding the potential effects of climate change on both the invasion ecology of the species and on the potential impacts of this species on the structure and function of invaded ecosystems.

We studied the sublethal effects of increased salinity at two temperatures to better understand the predicted niche of non-native blue catfish in coastal rivers of the eastern U.S. Specifically, we assessed differences in growth rates, body condition, consumption rates and proximate body composition—the relative proportions of water, lipids, protein and ash—of juvenile blue catfish exposed to one of four salinity treatments (1, 4, 7 or 10 psu) at either 12 or 22°C for 16 weeks. We hypothesized that fish growth, body condition and consumption rates would be maximized at intermediate salinities (4 or 7 psu) and 22°C. A salinity of 10 psu, however, was hypothesized to adversely impact blue catfish because individuals will need to adjust their osmoregulatory strategies in such hyperosmotic conditions [19].

## Methods

All animal capture, handling and experimental procedures were approved by the William & Mary Institutional Animal Care and Use Committee (protocols: IACUC-2016-08-19-11376-mcfabr and IACUC-2017-05-22-12111-tdtuck) and followed all applicable U.S. guidelines. Animal care was provided by the first author under the supervision of the second author. Both authors have 5+ years of experience handling fish in experimental and wild settings.

### Fish collections

Blue catfish (168–234 mm fork length [FL]) were captured from the tidal James River (coordinates 37°14’N 76°52’W) using a 9.14-m otter trawl following protocols of the Virginia Institute of Marine Science (VIMS) Juvenile Fish Trawl Survey; Tuckey and Fabrizio [7] provide details of the sampling design and protocols of this survey. Fish were collected from oligohaline reaches where salinity was <2 psu. This salinity threshold was chosen because few blue catfish of the desired size (<225 mm) are encountered at higher salinities; this observation is consistent with the reported relationship between fish size and salinity tolerance of blue catfish [5]. Blue catfish were brought to the VIMS Seawater Research Laboratory and treated prophylactically for potential parasites with a formalin bath and a saltwater dip using standard protocols [20]. To allow identification of individual fish, each fish was subsequently tagged with a unique 12.5 mm Passive Integrated Transponder (PIT) tag. After a three-day recovery period, blue catfish were randomly assigned to either the 12 or 22°C treatment group, and were acclimated for 3 weeks. During acclimation, salinity was 2 psu because preliminary trials showed high mortality of blue catfish at salinity ≤1 psu due to freshwater ich—a parasitic infection common to freshwater catfish species; ich infestations are impeded by chronic exposure to salinity >1 psu [20].

### Experimental setup

To study the combined effects of salinity and temperature, we used a 4×2 factorial design with four levels of salinity (1, 4, 7 and 10 psu) and two levels of temperature (12°C and 22°C); two replicate aquaria were maintained for each salinity-temperature treatment combination. For each temperature treatment level, we constructed two water baths, inside of which were randomly placed four identical 270-L cylindrical aquaria, corresponding to the four salinity levels. The experimental aquaria and the water bath exchanged heat but not water. The temperature of the water bath was controlled with an automated heater or chiller. We supplied each experimental aquarium with mechanical and biological filters and an aerator to maintain adequate dissolved oxygen concentrations (>6 mg O_2_/L at 22°C and > 8.5 mg O_2_/L at 12°C). To obtain the desired salinity levels, we diluted filtered York River water (mean salinity: 12.1 psu; range: 10.4–16.3 psu) with deionized water. Fish were fed commercial fish food (3 mm slow-sinking Finfish Silver; Zeigler Bros, Inc.) every other day *ad libitum* during the acclimation period and throughout the experiment; excess food and wastes were removed the next day. We monitored water quality (dO_2_, salinity, pH, NH_3_, NO_3_ˉ, and NO_2_ˉ) twice per week, and performed water changes as necessary to maintain water quality. The light schedule in the laboratory was computer-controlled to simulate natural photoperiod regimes, and all aquaria were partially covered to provide darkened areas for refuge.

We chose the salinity and temperature levels for the experiment based on a review of the literature. As the lowest salinity to be used for the experiment, we chose 1 psu to prevent ich infestations, as stated earlier. We chose 10 psu as the highest salinity treatment level because we assumed that long-term exposure to salinities greater than 9 psu (isosmotic salinity) would be energetically and osmotically too costly for fish and may lead to mortality [21]. We suspected that a salinity of 10 psu may lead to some osmotic stress but not mortality [5]. Blue catfish growth is maximized at 24°C [22], and suppressed at temperatures below 9°C [23]. We therefore chose 12 and 22°C as temperatures typical of areas of the Chesapeake Bay region occupied by blue catfish during the winter and spring (V. Nepal, *pers*. *obs*.).

The experiment was performed by exposing fish to 1 psu and subsequently increasing the salinity of the experimental aquaria at a fixed rate of 3 psu per day until target salinities were reached (*n* = 10 fish per aquarium). This rate of salinity increase is within the 1–5 psu per day range commonly used in similar studies [e.g., 24–28]. We held multiple fish in each aquarium because feeding declined considerably when only one individual was present (V. Nepal, *pers*. *obs*.). Fish were held in the aquaria for 16 weeks and checked once or twice a day for mortality. If a fish was unable to maintain equilibrium and exhibited reduced swimming ability or mouth gaping, the fish was considered moribund. Such fish were immediately removed from the trial and euthanized by immersion in an ice slurry and frozen for later analysis. All surviving fish were euthanized and frozen at the end of the experiment. Wet weights of all fish were recorded before freezing. At a later date, all frozen fish were processed to determine sex and obtain samples for subsequent analysis of proximate composition.

On day 71, all fish from two aquaria (salinity 10 psu, temperature 22°C, replicate 1, *n* = 10 fish; and salinity 1 psu, temperature 22°C, replicate 2, *n* = 10 fish) died of unknown causes. Water quality analyses and gross inspection of the dead fish revealed no abnormalities. These 20 fish were not included in mortality rate calculations, and were replaced with wild fish that had been maintained at 2 psu and 22°C and used for the remaining duration of the experiment. Fish were abruptly transferred to 1 psu, but salinity of the experimental aquarium at 10 psu was increased at the rate of 3 psu per day, as described above for other fish in this treatment group.

### Body size and condition

We recorded fork length (mm) and weight (0.1 g) of each fish at the beginning of the experiment and once every four weeks. Fish were not fed for 48 hours before length and weight measurements were recorded. We calculated relative condition factor (*K*_*n*_) as an index of body condition [29]. *K*_*n*_ > 1 implies higher condition than the average fish in the experiment, and *K*_*n*_ < 1 implies lower condition than the average fish in the experiment [29]. Sex of each blue catfish (male or female) was assessed at the end of the experiment by macroscopic examination of the gonads.

Changes in FL and *K*_*n*_ were analyzed using separate repeated measures analysis of variance models in the linear mixed-effects modeling framework. The models took the form:

$$Y_{ijklmn}\;=\;\mu +T_{j}+S_{k}+P_{l}+\;M{}_{m}+\beta B+a{}_{n}+\;f_{i(n)}+\varepsilon_{ijklmn}$$ (1)

where *Y*_*ijklmn*_ is the response variable (either FL or *K*_*n*_) for fish *i* (i.e., PIT tag *i*) from aquarium *n* in the temperature treatment *j* (12°C or 22°C), salinity treatment *k* (1, 4, 7 or 10 psu), measurement period *l* (4, 8, 12 or 16 weeks) and sex *m*; *μ* is the overall mean of the response *Y*; *T*_*j*_, *S*_*k*_, *P*_*l*_ and *M*_*m*_ are the fixed effects of temperature, salinity, measurement period and sex respectively; *β* is the regression coefficient for the effect of the baseline value of the response *B* (i.e., FL or *K*_*n*_ at the start of the experiment); *ε*_*ijklmn*_ is the unexplained random error assumed to have a normal distribution. The term *a*_*n*_ denotes the random effect of aquarium *n*, accounting for the potential pseudoreplication among observations from multiple individuals from a single aquarium. Similarly, *f*_*i*(*n*)_ denotes the random effect of fish *i* nested in aquarium *n*, accounting for the repeated measurements on each fish. We also included two- and three-way interactions among temperature, salinity and period. Our primary interest was in the interaction terms, which, if significant, would indicate significant diversions over time in FL or *K*_*n*_ at different temperatures (*T×P*), salinities (*S×P*) or both (*T×S×P*). The FL model included a two-way interaction between sex and time (*M×P*) to examine growth differences between males and females, because blue catfish show sexual dimorphism in growth patterns [30]. Other interaction terms were not considered because preliminary graphical analysis indicated lack of strong interactions. We used a first-order autoregressive (ar1) variance-covariance structure to account for the temporal autocorrelation in the response for each fish. Specifically, we used the heterogeneous ar1 structure because the variance increased over the measurement period. The Kenward-Roger method was used to calculate the degrees of freedom for the approximate *F*-tests.

Because change in FL over time (i.e., growth rate) was linear (see results below), we refit eq 1 for FL with period as a continuous predictor, and calculated *Q*_10_ for each salinity level to compare growth rates of blue catfish at different temperatures. We subsequently compared growth rates between temperatures and among salinities using bootstrap hypothesis tests [31]. Statistical analyses were conducted in SAS version 9.4 (SAS Institute, Cary, NC) following procedures in Stroup et al. [32].

### Body composition

We homogenized all blue catfish in an electric blender at the end of the experiment to assess differences in composition at different temperature-salinity combinations. Samples were dried at 60°C in a drying oven for several weeks. Once the sample had dried to constant weight, the tissue was homogenized further in a mortar and pestle and subsequently dried for another 48 hours. We calculated water content in each fish by subtracting the dry weight from the wet weight. Dry tissue samples were analyzed at the Aquaculture Laboratory in Southern Illinois University, Carbondale, Illinois, for proximate body composition. Fish dry tissues were separated into three components, namely lipids, protein and ash; carbohydrates were ignored because they form a minor constituent of fish tissues [17]. We report proximate body composition as fractional composition data where the four components (water, lipids, protein and ash) add up to 1. We were primarily interested in the relative ratios of components (e.g., lipid to ash ratio).

The components of compositional data such as ours must add to a constant, a condition called the constant-sum constraint, making traditional univariate or multivariate tests inappropriate [33]. We therefore analyzed the body composition data using Aitchison’s log-ratio approach [33]. Specifically, we transformed the four-part proximate body composition data into three transformed variables using the isometric log-ratio (ilr) transformation, which allows analysis of the transformed variables using classical statistical techniques [34]. The three transformed variables *z*_*water*_, *z*_*protein*_ and *z*_*lipids*_, called balances, were calculated as

$$z_{water}\;=\;\sqrt{\frac{3}{4}}\times \text{log}\;\frac{water}{\sqrt[{3}]{protein\times lipids\times ash}}$$ (2)

$$z_{protein}\;=\;\sqrt{\frac{2}{3}}\times \text{log}\;\frac{protein}{\sqrt{lipids\times ash}}$$ (3)

$$z_{lipids}\;=\;\sqrt{\frac{1}{2}}\times \text{log}\;\frac{lipids}{ash}$$ (4)

These ilr balances correspond to the ratio of water to all other components (*z*_*water*_), protein to the remaining components (*z*_*protein*_), and lipids to ash (*z*_*lipids*_), and can be back-transformed to proximate compositions to allow easy interpretation [34]. We modeled the ilr balances jointly using a multivariate linear mixed-effects model (LMM) of the form:

$$z_{cijkmn}\;=\;\mu +T_{j}+S_{k}+T_{j}\times S_{k}+M_{m}+\beta log\;(W)+a_{n}+\varepsilon_{ijkmn}$$ (5)

where *z*_*cijkmn*_ is the *c*^th^ ilr balance for fish *i* from aquarium *n* in temperature treatment *j*, salinity treatment *k* and sex *m*; *β* is the regression coefficient for the effect of natural log of fish weight log(*W*), and all other symbols are as described previously. We used log(*W*) instead of *W* because the former resulted in a better fit.

To ease interpretation, we obtained estimated marginal means for each balance at each salinity-temperature treatment combination, and back-transformed the marginal means to the four components (percent water, lipids, protein, and ash). We tested hypotheses of pairwise differences in mean proportions of each component among the salinity and temperature treatments using bootstrap techniques [31]. Specifically, we obtained 1,000 bootstrap resample datasets of ilr balances, with size of each bootstrap resample equal to the original sample size. We then fitted multivariate LMMs on each resample dataset and obtained the marginal means for each salinity-temperature treatment. Finally, we calculated bootstrap-based two-tailed *P*-values to compare statistically the estimated marginal means at different temperatures and salinities [31].

Treatment-specific differences in proximate body composition of blue catfish at the end of the experiment may result from differences that were present at the start of the experiment. To check for this potential confounding effect, we examined differences in proximate body composition of fish and wet weight of fish at the start of the experiment and between temperature treatments (12 or 22°C). To do this, we euthanized 30 randomly selected fish (*n* = 15 for each temperature level) before the start of experimental trials and obtained proximate body composition of these fish as stated above. We subsequently tested for the effects of fish weight and water temperature on mean body composition of these blue catfish using multivariate LMM of the form:

$$z_{cijm}\;=\;\mu +T_{j}+M{}_{m}+\beta log\;(W)+\varepsilon_{ijm}$$ (6)

where *z*_*cijm*_ is the *c*^th^ ilr balance for fish *i* of sex *m* held at temperature *j*; all other variables are as described above. A total of 210 fish were used in the experiment. Of these, 187 were euthanized, and 23 fish died before meeting the criteria for euthanasia (i.e., they died during the intervals between the routine checks).

We used the package robCompositions version 1.3.3 [35] in R version 3.6.1 (R Core Team, Vienna) for ilr transformation and back-transformation, and proc mixed in SAS to fit the multivariate LMM [36]. Assumptions of homogeneity of variance and normality of residuals were assessed using diagnostic plots.

### Consumption rate

In each experimental aquarium, we conducted feeding trials to determine the consumption rate of blue catfish at different salinities and temperatures. Due to logistical difficulties, we could not measure consumption rates of individual blue catfish; instead we measured the cumulative consumption rate for all (up to 10) blue catfish in each experimental aquarium. Fish were not fed for 48 hours before the consumption trials. A measured quantity of commercial fish feed was introduced to each experimental aquarium at 1700 hours, before the lights turned off. The fish were left undisturbed to allow feeding for the next 3 hours. We chose a relatively short period of 3 hours to minimize the accumulation of waste from egestion, while ensuring that blue catfish had enough time to consume the food. At 2000 hours, we transferred the uneaten food into pre-weighed aluminum pans, which were dried until constant weight at 60°C. The amount consumed (*C*_*F*_, g of food) was calculated by subtracting the dry weight of uneaten food from the weight of the food introduced in the corresponding aquarium. Consumption rate trials were conducted twice for each aquarium.

To adjust for effect of food disintegration on observed consumption rates, we conducted food disintegration trials in the experimental aquaria after the termination of the experiment when blue catfish were removed from the aquaria. We calculated the weight of food lost to disintegration (*C*_0_) after 3 h in each aquarium by subtracting the dry weight of remaining food from the weight of the food introduced in the aquarium. These amounts were calculated for each aquarium in the experiment and represented as *C*_0*i*_, or the amount of food lost to disintegration in aquarium *i*. The mass-specific consumption rates (*CR*, mg food per g fish) for each aquarium were subsequently calculated as:

$$CR_{ij}\;=\;\frac{C_{Fij}-C_{0i}}{\Sigma W_{i}}$$ (7)

where *CR*_*ij*_ is the mass-specific consumption rate for all blue catfish in aquarium *i* during event *j*, *C*_*Fij*_ is the amount of food consumed by blue catfish in aquarium *i* during event *j*, and Σ*W*_*i*_ is the total wet weight of blue catfish in aquarium *i*.

Effect of water temperature and salinity on the consumption rate of blue catfish was assessed using a generalized LMM of the form:

$${log\;(\lambda }_{jkn})\;=\;\mu +T_{j}+S_{k}+a_{n}+\varepsilon_{jkn}$$ (8)

where log(λ_*jkn*_) is the natural log of mean CR (λ) of all fish in aquarium *n* at temperature *j* and salinity *k*. Other variables are as before. Here, we used a gamma distribution with a log link, and fit the model using proc glimmix in SAS [32]. Some predictor variables were scaled to aid model fitting, and the intercept (*μ*) was suppressed to aid model interpretation. We report 95% confidence intervals (CI) for all predicted means and model parameters. Assumptions of homogeneity of variance and normality of residuals were assessed using diagnostic plots.

## Results

### Water quality

Water temperature and salinity were fairly stable during the experiment. Mean ammonia concentrations were higher during the first few weeks of the experiment, but decreased to acceptable low levels thereafter (S1 Table). Dissolved oxygen concentrations remained consistently high (> 5.0 mgL^-1^) in all aquaria, though values were lower for aquaria at 22°C (mean 7.4 mgL^-1^) than at 12°C (mean 10.8 mgL^-1^) due in part to reductions in oxygen solubility at higher temperatures. Mean pH of all aquaria was 7.4 with little fluctuation (S1 Table).

### Survival, body size and condition

**Fig 1:**
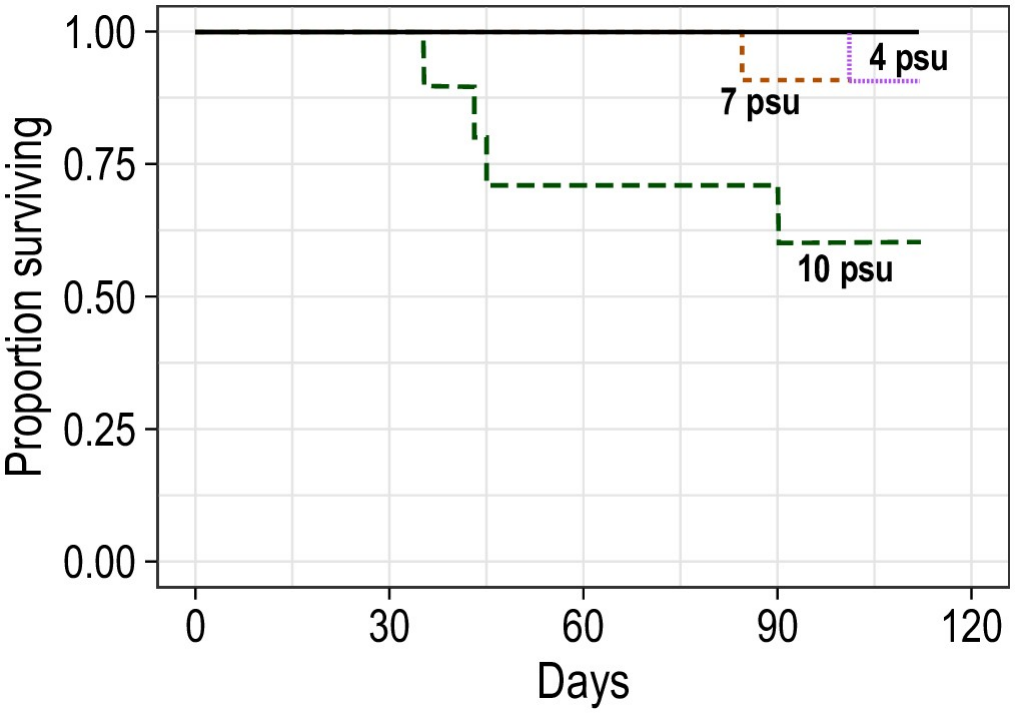
Survival of blue catfish over time in various salinity treatments at 12 and 22°C for 112 days. Each line represents one aquarium with 10 blue catfish; black solid line includes multiple overlapping lines. All mortalities occurred in 22°C treatments.

Of the 160 experimental fish, 154 (96.25%) survived to the end of the experiment. Six fish that died during the experiment were in the 22°C treatments: four fish died at 10 psu (20% mortality rate), and one fish died in each of the 7 and 4 psu treatments (5% mortality rate; Fig 1).

**Fig 2:**
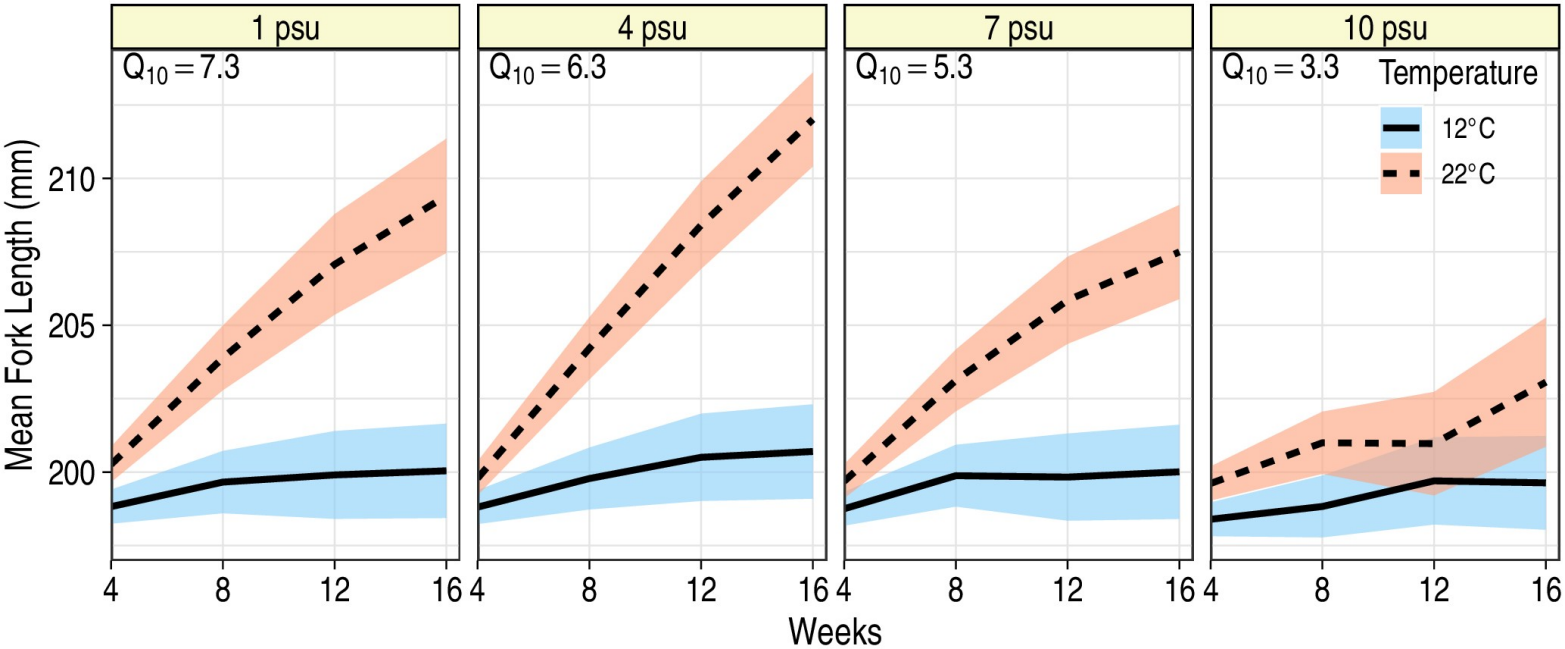
Mean fork length of juvenile blue catfish during a 16 week period at two temperatures and four salinities. Ribbons represent 95% confidence bands around the predicted mean fork lengths. Predictions are for a fish that was 198 mm at the start of the experiment (i.e., at week 0). For each salinity, Q_10_ estimates, assuming linear growth, are provided at the top left corner of each panel.

Temperature had a positive effect on growth rate of juvenile blue catfish: growth rates were faster at 22°C than at 12°C (*P* < 0.05; Table 1; Fig 2). There was, however, an interactive effect of time with temperature and salinity (*F*_3,198_ = 11.1; *P* < 0.001) reflecting differences in growth patterns among the treatment groups. Pairwise comparisons revealed that growth rates at 12°C were similar across salinity levels (*P* > 0.999), but at 22°C, considerable differences existed such that fish grew fastest at 4 psu and slowest at 10 psu (Fig 2, Table 2). Variance in FL measurements increased over time, and proximal FL measurements on the same fish were more correlated than measurements further apart in time (Table 3). *Q*_10_’s at 1, 4, 7 and 10 psu were 6.8, 6.0, 5.1 and 3.2, respectively, implying that increased temperature had the greatest positive impact on blue catfish at 1 psu and smallest positive impact on fish at 10 psu (Fig 2). Unsurprisingly, initial size of the fish was highly predictive of subsequent FL measurements (*F*_1,144_ = 26663.6, *P* < 0.001), indicating that through time, larger fish continued to be larger than their smaller counterparts. Furthermore, we found no evidence for sexual dimorphism in growth rates of blue catfish (*F*_1,186_ = 0.09; *P* = 0.763).

**Table 1:** Bootstrap-based *P*-values comparing growth rate (change in FL/day), proportions of water, protein, lipids and ash, and consumption rates of juvenile blue catfish at 12 versus 22°C at 1, 4, 7 or 10 psu.

Salinity (psu)	Growth rate	Prop. water	Prop. protein	Prop. lipids	Prop. ash	Consumption rate
1 psu	**<0.001**	**0.008**	0.730	**0.048**	0.922	0.074
4 psu	**<0.001**	**<0.001**	0.188	0.172	**0.004**	0.121
7 psu	**<0.001**	**0.014**	0.738	0.110	0.364	**0.022**
10 psu	**0.050**	0.652	0.362	0.326	0.610	**0.046**

*P*-values < 0.05 are shown in bold.

**Table 2:** Bootstrap-based *P*-values comparing pairwise differences in growth rate (change in FL/day), proportions of water, protein, lipids and ash, and consumption rates of juvenile blue catfish at 1, 4, 7 or 10 psu at 12 or 22°C.

Temperature	Comparison	Growth rate	Prop. water	Prop. protein	Prop. lipids	Prop. ash	Consumption rate
12°C	1 psu v 4 psu	>0.999	0.716	0.546	0.494	0.56	>0.999
	1 psu v 7 psu	>0.999	0.444	0.338	0.294	0.842	0.680
	1 psu v 10 psu	>0.999	0.682	**0.038**	0.144	0.532	0.064
	4 psu v 7 psu	>0.999	0.636	0.592	0.592	0.448	>0.999
	4 psu v 10 psu	>0.999	0.470	**0.002**	0.054	0.870	0.479
	7 psu v 10 psu	>0.999	0.268	**0.006**	**0.014**	0.436	>0.999
22°C	1 psu v 4 psu	0.121	0.110	0.618	0.720	**0.014**	>0.999
	1 psu v 7 psu	**<0.001**	0.396	0.290	0.268	0.252	>0.999
	1 psu v 10 psu	**<0.001**	**<0.001**	**0.040**	**<0.001**	0.280	0.091
	4 psu v 7 psu	**<0.001**	0.424	0.186	0.654	0.110	>0.999
	4 psu v 10 psu	**<0.001**	**<0.001**	0.232	**<0.001**	**<0.001**	>0.999
	7 psu v 10 psu	**<0.001**	**<0.001**	**0.002**	**<0.001**	0.052	0.777

*P*-values < 0.05 are shown in bold.

**Table 3:** Random effects parameter estimates for mixed effects models fitted on fork length (FL) or body condition (*K*_*n*_) of blue catfish exposed to increased salinity at 12 or 22°C.

Parameter	Estimate for FL	Estimate for *K*_*n*_
*σ*^2^_week 4_	1.48	0.0013
*σ*^2^_week 8_	4.97	0.0031
*σ*^2^_week 12_	9.85	0.0033
*σ*^2^_week 16_	11.3	0.0038
*ρ*	0.84	0.6993

*σ*^2^ = variance; *ρ =* correlation

**Fig 3:**
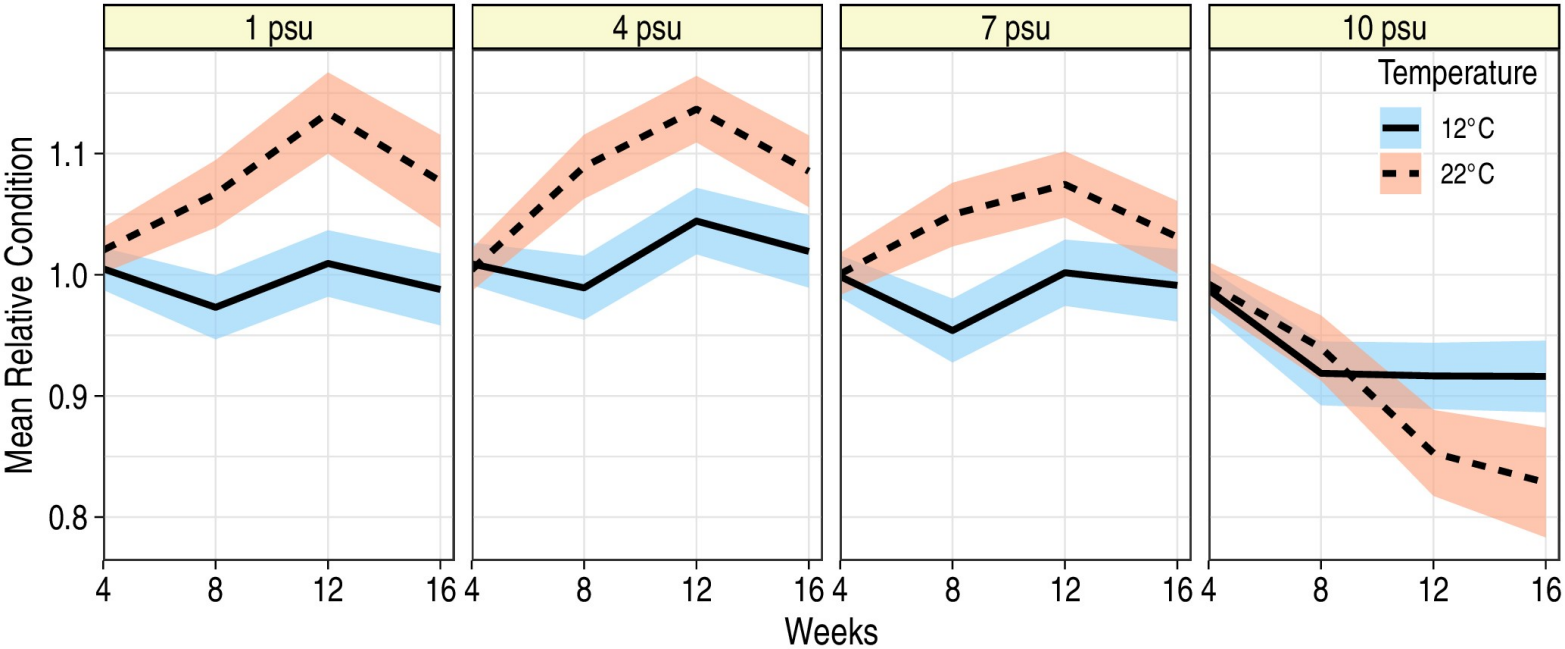
Mean relative condition factor (*K*_*n*_) of juvenile blue catfish during a 16 week period at two temperatures and four salinities. Ribbons correspond to 95% confidence bands around the predicted mean condition factors. Predictions are for a fish that had a *K*_*n*_ of 1.02 at the start of the experiment (i.e., at week 0).

Mean body condition exhibited a significant interaction among time, temperature and salinity (*F*_9,308_ = 7.25; *P* < 0.001), however, at salinities of 7 or less, temperature had a largely positive effect on mean body condition with significantly higher *K*_*n*_ at 22°C than at 12°C (Fig 3). At 12°C, mean *K*_*n*_ at 1, 4 or 7 psu was fairly stable through time, fluctuating around the mean of 1.0; at 22°C, mean *K*_*n*_ increased through time for fish in the 1, 4 and 7 psu treatment levels. These patterns were different for fish held at 10 psu: mean body condition declined for fish at both 12 and 22°C, with the most severe declines observed at 22°C (Fig 3). Repeated measurements of the same fish revealed that fish at 10 psu, and in particular those at the 10 psu-22°C treatment, were also less able to heal skin abrasions. Similar to FL, variance in *K*_*n*_ measurements increased over time, and proximal *K*_*n*_ measurements on the same fish were more correlated than the measurements taken further apart in time (Table 3). In general, fish with high mean initial *K*_*n*_ continued to exhibit high mean *K*_*n*_ throughout the experiment (*F*_1,138_ = 430.3; *P* < 0.001); sex did not affect *K*_*n*_ (*F*_1,138_ = 0.82; *P* = 0.37).

### Body composition

**Fig 4:**
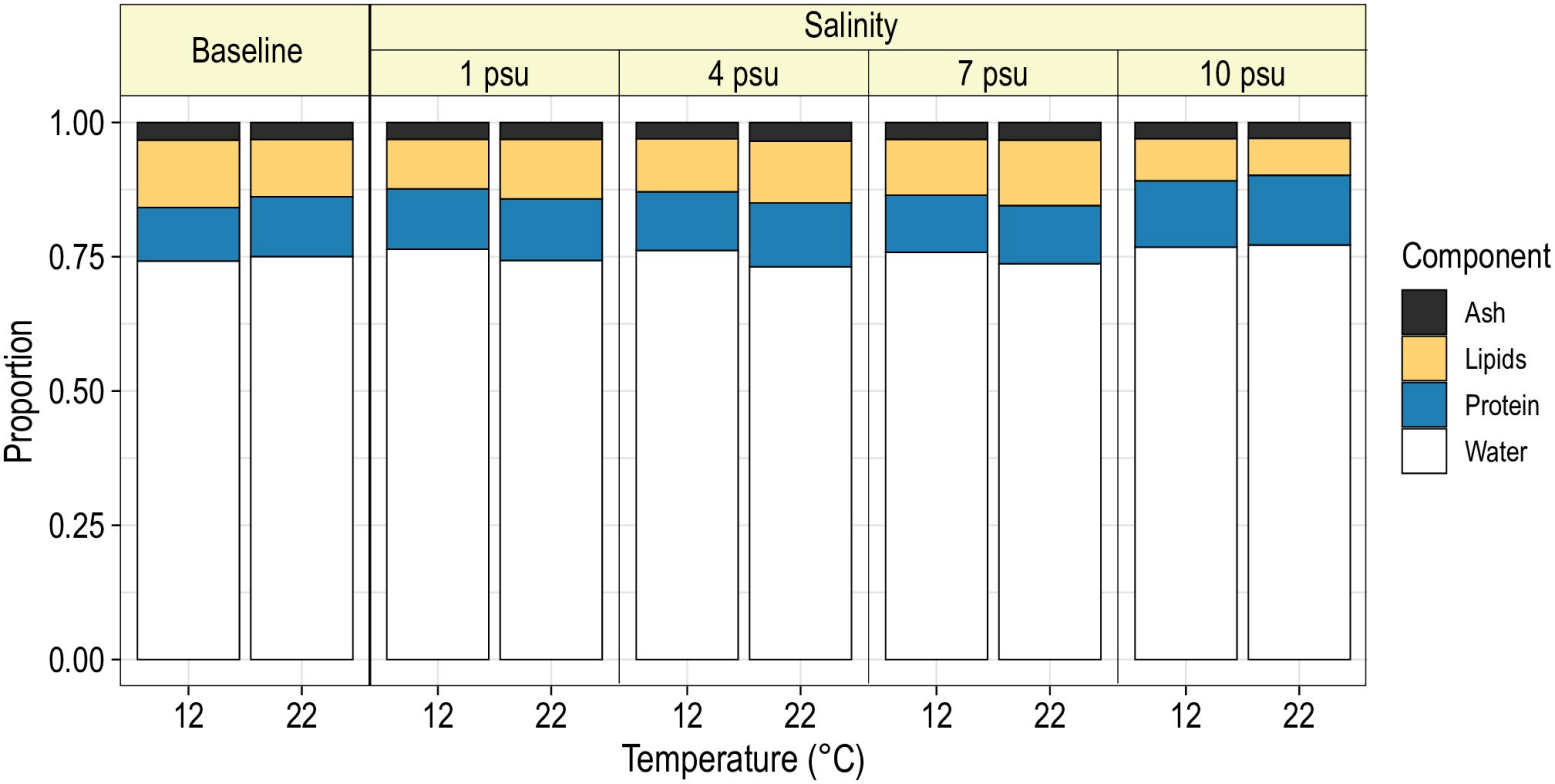
Mean body composition of juvenile blue catfish subjected to one of four salinities at 12 or 22°C for 16 weeks. Baseline refers to mean body composition of blue catfish prior to exposure to salinity treatments. Predictions are for a fish with wet weight of 96.5 g (average weight of fish in the analysis).

On average, water, protein, lipids and ash comprised 74.5%, 14.8%, 7.2% and 3.6% of the wet weight. However, mean relative proportions of these components differed considerably among treatment levels and between the initial and post-experimental period. Blue catfish at 12°C that were sacrificed before the start of the experiment had significantly different mean body compositions than fish at 22°C (*F*_3,30_ = 6.3; *P* = 0.002). Specifically, compared with the fish at 12°C, fish at 22°C had a significantly greater mean proportion of protein (bootstrap *P* < 0.001), but a significantly lower mean proportion of lipids (bootstrap *P* = 0.036); mean proportions of water and ash did not differ significantly between fish from the two temperatures (Fig 4). Mean body compositions were not significantly affected by initial wet weight (*F*_3,30_ = 2.15; *P* = 0.115) or sex (*F*_3,30_ = 1.19; *P* = 0.331).

**Fig 5:**
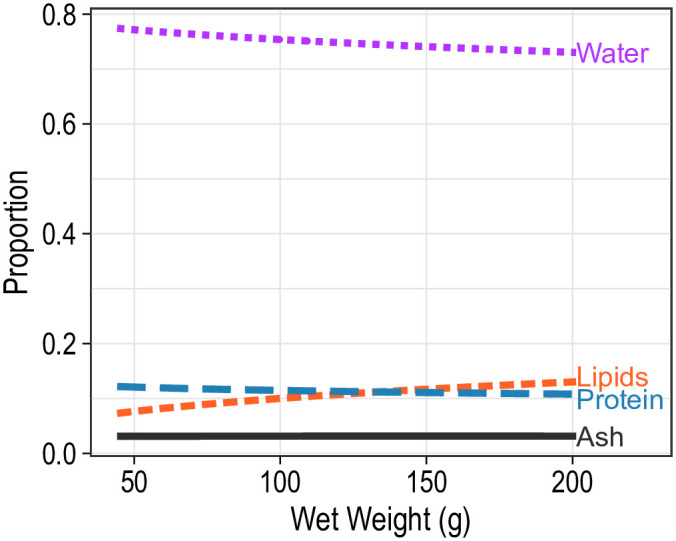
Mean body composition of juvenile blue catfish as a function of wet weight of the fish.

Mean body composition of fish differed significantly with temperature (*F*_3,424_ = 7.5; *P* < 0.001) and salinity (*F*_9,424_ = 3.4; *P* < 0.001), and the interaction between temperature and salinity was not significant (*F*_9,424_ = 1.45; *P* = 0.163). Bootstrap analysis revealed that the mean proportion of water was significantly higher for fish at 12°C than at 22°C at 1 (*P* = 0.008), 4 (*P* < 0.001) and 7 psu (*P* = 0.014; Table 1; Fig 4). Within the 12°C treatment, mean proportion of protein was significantly lower for blue catfish held at 10 psu compared with fish in lower salinities (*P* < 0.05; Table 2; Fig 4). Most other components did not differ significantly among the salinity treatment levels. Similarly, at 22°C, the primary differences were observed between fish at 10 psu and those in lower salinities: fish at 10 psu had significantly higher mean proportions of water and lower mean proportions of lipids than those at 1, 4 or 7 psu (*P* < 0.05; Table 2; Fig 4). Mean body compositions were similar for males and females (*F*_3,424_ = 1.4; *P* = 0.242) but differed significantly with fish weight (*F*_3,424_ = 7.5; *P* < 0.001). As fish increased in length, the mean proportion of water decreased and the mean proportion of lipids increased, but the mean proportions of protein and ash remained stable (Fig 5).

### Consumption rate

**Fig 6:**
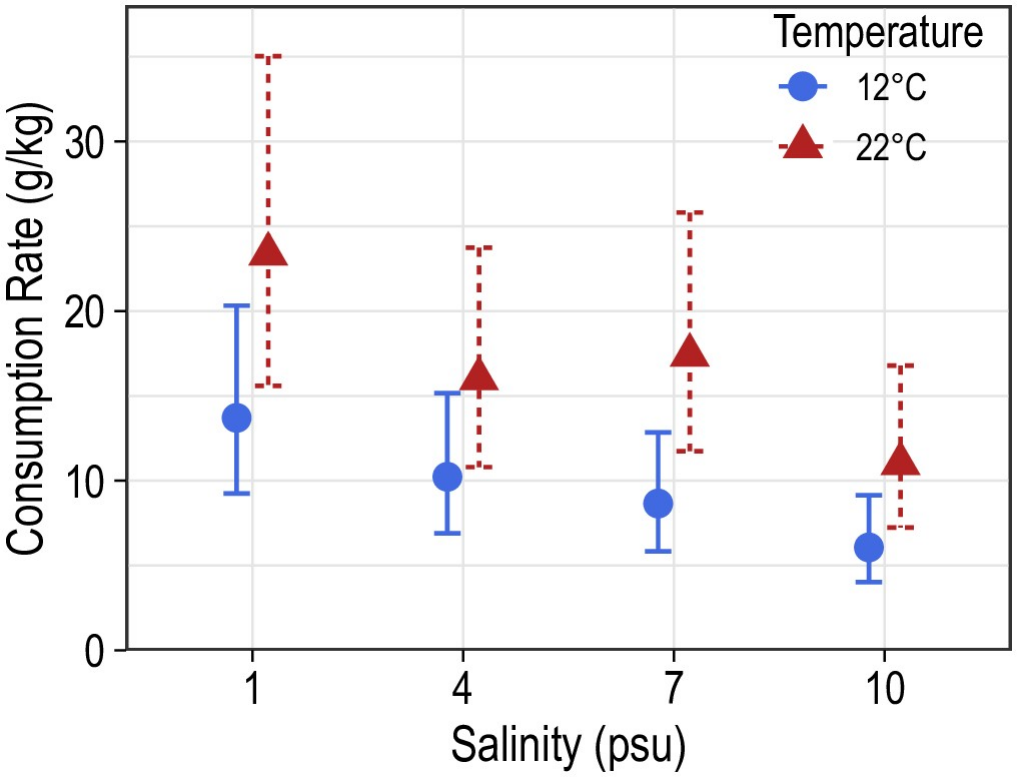
Mean consumption rates (g/kg) of juvenile blue catfish at two temperatures and four salinities. Error bars correspond to 95% confidence bands around the predicted consumption rates.

Consumption rates of blue catfish ranged between 3.5 and 35.0 g/kg of fish body weight and varied considerably within aquaria (intraclass correlation = 0.12). Mean consumption rates were highest at 1 psu and 22°C (23.4 g/kg of the fish body weight) and lowest at 10 psu and 12°C (6.1 g/kg of the fish body weight). Temperature had a significant positive effect on consumption rates (*F*_1,16_ = 17.2; *P* < 0.001; Fig 6), though these differences were significant only at 7 (*t*_16_ = 2.6; *P* = 0.022) and 10 psu (*t*_16_ = 2.2; *P* = 0.046; Table 1). Increased salinity negatively influenced mean consumption rates (*F*_3,16_ = 5.2; *P* = 0.011; Fig 6), however, pairwise comparisons did not reveal significant differences in mean consumption rates among salinities within a temperature (Table 2).

## Discussion

Most juvenile blue catfish in the Chesapeake Bay region can survive in salinities up to 10 psu for 112 days. Salinities up to 7 psu seemed to have little negative impact on growth, body condition and compositions. Together with previous research that demonstrated high short-term tolerance of blue catfish to acute changes in salinity [5], these findings suggest that U.S. Atlantic coast habitats with salinities ≤7 psu are vulnerable to establishment of blue catfish populations. In these habitats, blue catfish may negatively impact local estuarine animals via competition and predation. Blue catfish may also use brackish-water environments to alleviate density-dependent intraspecific competition experienced by conspecifics in freshwater environments and to disperse to previously uninvaded rivers. Further, higher temperatures had positive effects on blue catfish at salinities ≤7 psu. As such, increases in winter and spring water temperatures due to global warming may foster establishment in these brackish water habitats.

Salinity and temperature had an interactive effect on blue catfish biology, in agreement with reports for other species (e.g., grass carp *Ctenopharyngodon idella* [24, 37]; Atlantic cod *Gadus morhua* [38]; Nile tilapia *Oreochromis niloticus* [39]). In general, blue catfish had higher consumption rates, faster growth, better body condition, and a greater proportion of lipids at 22°C than at 12°C. Higher consumption and growth rates of animals at higher temperatures is a well-known tenet in physiology (e.g., [17]). Further, the greater proportion of lipids and lower proportion of water and ash in fish held at high temperatures likely indicate faster short-term growth [40].

While positive effects were observed with increases in temperature at salinities ≤7 psu, this was not the case at 10 psu, where mean growth rates and body conditions declined significantly at the higher temperature. In particular, fish at the 10 psu-22°C treatment were emaciated (i.e., low *K*_*n*_), less able to heal abrasions and had lower mean proportions of lipids compared with fish from other treatments. These results conform to expectations from osmoregulatory physiology, emphasizing that the physiological mechanisms in freshwater fish are unable to maintain homeostasis in hyperosmotic environments (i.e., >9 psu; [21, 41]). As such, these fish allocated less energy to growth (both in terms of length and mass) and had low lipid reserves. Many other studies reported similar results where growth rates and body condition of freshwater fishes decline starkly at salinities greater than ~9 psu (e.g., channel catfish *Ictalurus punctatus* [42], goldfish *Carassius auratus* [28]; feral catfish *Heterobranchus bidorsalis* [43]; Asian swamp eel *Monopterus albus* [26]).

The optimal salinity for juvenile blue catfish appears to be around 4 psu as indicated by fastest growth and good body condition despite relatively low mean consumption rates. These results support a previous study on larval blue catfish which reported the highest survival and growth at 3 psu [15] and other studies of freshwater species (e.g., freshwater snakehead *Channa punctata* 5 psu [44], and Asian swamp eel 3 psu [26]). This may be because the osmotic gradient is lower at these salinities, and hence smaller amounts of energy are spent on osmoregulation, leaving a larger fraction of energy for growth [21, 41]. Others have found contrasting results for freshwater fish with fastest growth in freshwater (e.g., [25, 28]), or similar growth rates up to the isosmotic salinity (e.g., [42, 43]). Consumption rates of freshwater fishes can also increase with salinity (e.g., [45]), decrease with salinity (e.g., [46]) or maximize at intermediate salinities (e.g., [27]). Taken together, the effects of salinity on freshwater fishes appear to be species-specific.

Salinities at the sampling locations and during the acclimation period could have affected our results. Salinity at the sampling location fluctuates daily and seasonally between 0 and ~4 psu. Because exposure to low or moderate salinity levels often upregulates hyperosmotic abilities in fish [47], the osmotic ability of our experimental fish was likely already upregulated before the experiment. Therefore, we are confident that our results are relevant to most wild blue catfish in brackish water habitats in the eastern U.S. Yet, our results may not hold for larger fish—which have greater acute salinity tolerance [5], or for fish that have never been exposed to brackish waters—e.g., blue catfish in their native freshwater habitats in midwestern rivers.

The observed effects of salinity and temperature may result from any of several proximate physiological modes of action, including changes in consumption rate (i.e., food detection ability or appetite), assimilation rate, or the partition of assimilated energy to various life processes such as maintenance of homeostasis, activity, and somatic or gonadal growth [48]. In particular, decreases in consumption rates in brackish waters may be a result of reduced prey detection ability due to the diminished electroreceptory ability of blue catfish in brackish waters. Catfishes of the order siluriformes are electroreceptive, and can use electroreception for prey detection [49]. Electroreceptory organs in freshwater fishes, however, are anatomically different from those in saltwater species, and thus, do not function in brackish and marine waters [49]. It seems likely, therefore, that blue catfish may not be able to detect prey as well in brackish waters, leading to lower consumption rates. We cannot, however, rule out other potential modes of action, particularly because multiple modes of action likely act concurrently. For example, compared with fish at 1 psu, fish at 4 psu may feed less, and have a lower assimilation rate, but still maintain high growth by allocating a smaller fraction of energy to maintenance of osmoregulatory homeostasis. The specific combinations of these modes of action that lead to specific response of fishes to changing salinity are likely to depend on the evolutionary history and life-history adaptations of the species. This is evidenced by the observation that even though most freshwater fishes are relatively uncommon in estuaries, some groups of freshwater fishes, such as members of the family Cichlidae, have unusually high salinity tolerance and occupy a wide range of estuarine and marine environments [10].

Future research should attempt to identify the combination of modes of action that lead to the observed results, though bioenergetics modeling may also reveal likely processes [48]. Towards this end, our results provide important inputs for the parameterization of a bioenergetics model that accounts for the effect of salinity and temperature on vital rates of blue catfish in coastal rivers. The inferences from our study and their use in bioenergetics modeling would have benefitted from measurements of consumption rates, egestion rates and energy assimilation rates at the level of individual fish. Measurement of these rates at finer resolutions of temperature, and especially salinity, could also help obtain a better understanding of the effects of temperature and salinity on blue catfish.

Despite the suggestion from our results that brackish water habitats with salinities ~4 psu provide the most energetically optimal environments for blue catfish, >45% of blue catfish captured from the tidal James and York rivers in the Chesapeake Bay by a fishery-independent trawl survey (VIMS trawl survey) occurred at salinities ≤1 psu [5]. Given the general observation that estuarine and marine environments have higher food levels [10], this discrepancy suggests that salinities >1 psu may have negative impacts on other aspects of blue catfish biology not studied here. For example, Perry [50] suggested that reproduction of blue catfish may be curtailed at salinity >2 psu, though it is not clear whether this is caused by hinderance in development of oocytes or mortality of eggs and larvae. Maternal effects (e.g., increased salinity tolerance of the offspring from mothers pre-exposed to increased salinities [51]) and behavioral effects (e.g., decreased parental care of eggs from fathers under high salinity conditions [52]) may also play important roles, but have not been studied. Research is needed to estimate sublethal effects on the reproductive biology of blue catfish. We note that the reproductive biology of blue catfish has been described in two invasive populations [53], and that because these systems are tidally influenced, an individual fish can potentially use salinities >2 psu for foraging and dispersal or to offload parasitic infestations yet return to freshwater habitats for spawning.

Relatively high tolerance to acute [5] and chronic exposures to increased salinities suggest that blue catfish are able to establish in many brackish-water habitats throughout the Eastern U.S. High salinity tolerance has been suggested as an important trait allowing invasion of and range expansion via estuarine and coastal habitats by many freshwater species including several catfishes (e.g., suckermouth armored catfish *Pterygoplichthys* spp. [54], flathead catfish *Pylodictis olivaris* [16], channel catfish [55]) and European catfish *Siluris glanis* [56]). As a novel, generalist omnivore in estuarine habitats, blue catfish may negatively impact the native estuarine organisms indirectly through habitat modification as well as directly through predation and competition. For example, Schmitt et al. [57] reported an increase in predation on native blue crabs *Callinectes sapidus* by blue catfish at higher salinities. Focused studies assessing the diet of blue catfish in high salinity habitats are needed to understand impacts on other estuarine animals of economic or cultural value. We predict that the overall negative impacts of individual blue catfish on local fauna at salinities >2 psu may not be high because of relatively lower consumption rates in brackish environments compared with freshwater environments. Yet, the total impacts may be high if the population size of the fish at these salinities is high. Even though such areas are not likely to support reproduction, they are likely to support foraging and dispersal, potentially allowing blue catfish to form metapopulations.

Our study provides an indication of the fundamental niche of blue catfish in relation to the salinity and temperature axes (*sensu* [9]) and provides crucial information towards development of a mechanistic species distribution model [58] for blue catfish throughout tidal rivers and estuaries of the U.S. Atlantic slope. These findings also emphasize the need to consider multiple biological end-points (e.g., growth, body condition, body composition) and to consider important environmental variables together when studying their effects on fish biology as experiments that incorporate factorial designs are likely to yield more realistic predictions than more simplistic experiments that focus on a single variable. Overall, our results indicate that estuarine habitats throughout the Eastern U.S. with salinities ≤7 psu are vulnerable to blue catfish establishment, and thus critical habitats at these salinities (e.g., areas that provide nursery habitats for species of conservation concern) could be prioritized for protection by state and regional management agencies. Down-estuary shift of salinity gradients during wet years or increased water temperatures due to global warming are likely to increase the chances of dispersal, range expansion [5], and establishment of blue catfish, and hence the severity of its impacts in brackish-water habitats. On the other hand, salinity intrusion with sea level rise, as predicted to occur in coastal and estuarine waters, may serve to limit dispersal pathways and lead to formation of discrete subpopulations of blue catfish that are intermittently connected during periods of high flow.

## Supporting information

S1 Checklist (DOCX)

S1 Data (XLSX)

S1 Table Water quality variables measured in the experimental aquaria where blue catfish were exposed to one of four salinities at either 12 or 22°C for a period of 16 weeks. Values of the water quality represent the mean ± SEM. psu = practical salinity units; dO_2_ = Dissolved Oxygen.
(DOCX)
