# Supplementary material for: Sublethal effects of salinity and temperature on non-native blue catfish: Implications for establishment in Atlantic slope drainages
Source: PLoS One. 2020 Dec 29;15(12):e0244392. doi: 10.1371/journal.pone.0244392 (PMC7771670; doi:10.1371/journal.pone.0244392)
Supplement: S1 Table — Values of the water quality represent the mean ± SEM. psu = practical salinity units; dO2 = Dissolved Oxygen. (DOCX) [file pone.0244392.s003.docx]

**Nepal and Fabrizio: Sublethal effects of salinity and temperature on non-native blue catfish: implications for establishment in Atlantic slope drainages**

**S1 Table: Water quality variables measured in the experimental aquaria where blue catfish were exposed to one of four salinities at either 12 or 22°C for a period of 16 weeks.** Values of the water quality represent the mean ± SEM. psu = practical salinity units; dO_2_ = Dissolved Oxygen

| Temperature (°C) | Salinity (psu) | Ammonia (mg/L) | dO_2_ (mg/L) | pH |
| --- | --- | --- | --- | --- |
| 12 | 1 | 0.3±0.1 | 10.9±0.3 | 7.2±0.1 |
| 12 | 4 | 0.5±0.2 | 10.2±0.3 | 7.2±0.1 |
| 12 | 7 | 0.6±0.2 | 10.8±0.2 | 7.3±0.1 |
| 12 | 10 | 0.5±0.2 | 10.9±0.1 | 7.4±0.1 |
| 22 | 1 | 0.8±0.3 | 7.7±0.2 | 7.4±0.1 |
| 22 | 4 | 0.2±0.1 | 7.7±0.2 | 7.4±0.1 |
| 22 | 7 | 0.2±0.1 | 7.0±0.1 | 7.5±0.0 |
| 22 | 10 | 0.5±0.1 | 7.2±0.1 | 7.4±0.1 |
